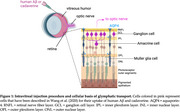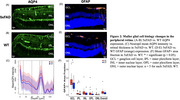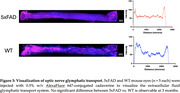# Retinal Müller Glia Alterations and Ocular Glymphatic Clearance in an Alzheimer's Disease Mouse Model

**DOI:** 10.1002/alz70855_103984

**Published:** 2025-12-24

**Authors:** Glori Das, Raksha Raghunathan, Lin Wang, Zhihao Wan, Hong Zhao, Stephen T.C. Wong

**Affiliations:** ^1^ Houston Methodist Research Institute, Houston, TX, USA

## Abstract

**Background:**

Amyloid beta (Aβ) deposits are well‐documented in the retinas of Alzheimer's disease (AD) patients, with retinal Aβ levels closely correlating with brain Aβ deposition. Impaired glymphatic clearance is a key mechanism implicated in Aβ accumulation in the AD brain; however the potential role of glymphatic clearance deficits in driving retinal Aβ accumulation remains poorly understood. Furthermore, alterations in Müller glial cell (MGC) biology, known to play a critical role in retinal homeostasis and neuroinflammatory responses, may exacerbate Aβ pathology. This study investigates whether optic nerve glymphatic clearance impairments and Müller glia cell (MGC) biology alterations collectively contribute to retinal Aβ accumulation.

**Methods:**

Comparing 3‐month‐old female 5xFAD vs. wild‐type (WT) mice, we compared MGC phenotypic differences and optic nerve glymphatic clearance rates. Immunofluorescence was used to quantify MGC expression of glial fibrillary acidic protein (GFAP) to quantify gliosis and aquaporin‐4 (AQP4), which is the main molecular driver of glymphatic clearance in both the brain and the eye. To observe glymphatic clearance in the anterograde direction (from eye toward brain), we performed intravitreal injections of fluorescent human Aβ40 as well as fluorescent cadaverine to observe extracellular fluid transport.

**Results:**

AQP4 demonstrated a robust upregulation across all retina layers in 5xFAD mice, marked by enhanced perivascular localization and increased expression in the neuropil. GFAP levels were notably elevated in the peripheral retina of 5xFAD mice, suggesting potential reactive gliosis and glymphatic disruptions originating in these regions. Importantly, the study observed consistent anterograde glymphatic transport of fluorescent Aβ or cadaverine tracers via the optic nerve, indicating maintained transport efficiency in this pathway.

**Conclusions:**

These findings reinforce and expand on prior evidence that Müller glial cell (MGC) biology undergoes significant changes in the early stages of AD, potentially preceding clinical symptoms. The identification of the peripheral retina as a critical yet underexplored region in understanding retinal AD pathology highlights its potential as an accessible biomarker and a potential target for early intervention. Further research will dissect the complex interplay between local Aβ production and clearance to clarify the retina's role in AD progression, paving the way for novel diagnostic and therapeutic strategies.